# Fueling recovery: The importance of energy coupling between angiogenesis and osteogenesis during fracture healing

**DOI:** 10.1016/j.bonr.2024.101757

**Published:** 2024-03-25

**Authors:** Fleur van Brakel, Yudong Zhao, Bram C.J. van der Eerden

**Affiliations:** Calcium and Bone Metabolism Laboratory, Department of Internal Medicine, Erasmus MC, Erasmus University Medical Center, Rotterdam, the Netherlands

**Keywords:** Fracture healing, Bone regeneration, Angiogenesis, Osteogenesis, Energy metabolism, Metabolic coupling

## Abstract

Approximately half of bone fractures that do not heal properly (non-union) can be accounted to insufficient angiogenesis. The processes of angiogenesis and osteogenesis are spatiotemporally regulated in the complex process of fracture healing that requires a substantial amount of energy. It is thought that a metabolic coupling between angiogenesis and osteogenesis is essential for successful healing. However, how this coupling is achieved remains to be largely elucidated. Here, we will discuss the most recent evidence from literature pointing towards a metabolic coupling between angiogenesis and osteogenesis. We will describe the metabolic profiles of the cell types involved during fracture healing as well as secreted products in the bone microenvironment (such as lactate and nitric oxide) as possible key players in this metabolic crosstalk.

## 1 Fracture healing

Upon injury, bone tissue has the impressive capacity to regenerate without forming a fibrous scar. Fractures can heal via two processes: direct healing and indirect healing. Direct healing includes the process of intramembranous ossification, whereby mesenchymal stromal cells (MSCs) directly differentiate into bone-forming osteoblast (Bahney et al., 2019; Kostenuik and Mirza, 2017). Indirect healing involves a cartilage intermediate, which is formed by the process of endochondral ossification, prior to bone formation by osteoblasts. Directly following injury, when the blood vessels rupture and hemorrhage, the process of fracture healing initiates with hematoma formation (Marsell and Einhorn, 2011). The lack of circulation, ischemia, leads to less oxygen supply in the fracture callus. Immediate effects of traumatic injury on tissue oxygen levels at the fracture site were observed in mice, with the mean pO2 at the fracture site being significantly lower compared to the periosteum in contralateral limbs. In case of prolonged ischemic insults, such as femoral artery resection, this injury becomes severe enough to delay fracture healing (Lu et al., 2008). Insignificant oxygen supply is often accompanied by acidosis due to reduced blood flow and increased glycolysis, leading to local hypoxia, lower pH, and higher lactate levels in the healing callus (Spector et al., 2001). Subsequently, the inflammatory response leads to the invasion of immune cells and the coagulation of the hematoma, forming the foundation of the callus (Marsell and Einhorn, 2011). Hereafter, the fibrovascular phase is initiated (Bahney et al., 2019). In this phase, new blood vessels are formed by the process of angiogenesis, and MSCs are recruited to the fracture site, which eventually differentiate into fibroblasts, chondroblasts and osteoblasts. From there on, a fibrocartilaginous callus is formed, which will be converted in spongy bone and will eventually be remodeled by osteoclasts and osteoblasts into compact bone (Kostenuik and Mirza, 2017). A small part of the fractures shows impaired healing, referred to as delayed union or non-union (Ding et al., 2018). This can be accounted for by various reasons, such as age, systemic diseases or poor mechanical stability. In up to 46 % of the impaired tibia fracture healing cases, the blood supply is insufficient (Dickson et al., 1995). Therefore, it is essential to know how angiogenesis is initiated during fracture healing and how this process is coupled to bone regeneration.

## 2 The angiogenic-osteogenic interaction

The process of angiogenesis is essential for successful fracture repair. Due to blood vessel rupture, there is a lack of vascular supply to the fracture site, resulting in local necrosis, acidosis and hypoxia. Transcription factor hypoxia-inducible factor 1-alpha (HIF1α) is stabilized in hypoxic conditions in endothelial cells (ECs) (Tao et al., 2022). This leads to the release of vascular endothelial growth factor (VEGF), which causes recruitment and proliferation of endothelial progenitor cells to the fracture site, driving angiogenesis. Osteoblasts also play a crucial role in angiogenesis during fracture repair by the production of VEGF as short-term fracture-induced hypoxia upregulates osteoblast VEGF expression (Tao et al., 2022). However, it is important to mention that extreme environmental stress in the ischemic fracture microenvironment hampers osteoblasts' ability to recruit new blood supply. If hypoxic conditions persist, increasing concentrations of anaerobic metabolism products, including lactate and hydrogen ions, blunt VEGF expression in osteoblasts (Spector et al., 2001). The hypoxic environment does not only have an impact on angiogenesis, but also on the process of osteogenesis. The activation of HIF1α under hypoxia in osteoblasts, but also paracrine VEGF signaling, promotes the osteogenic differentiation of MSCs (Yu et al., 2019; Hu and Olsen, 2016). Indeed, it has been shown that HIF1α knockout mice show reduced vessel formation and decreased bone regeneration compared to control mice (Wang et al., 2007; Tomlinson and Silva, 2015), suggesting an overlap in activation of angiogenesis and osteogenesis via HIF1α activation and VEGF signaling. According to these results, it is implicated that the processes of angiogenesis and osteogenesis are closely coupled in a spatiotemporal manner. This bidirectional crosstalk can be achieved via the aforementioned paracrine signaling, which was investigated with condition medium experiments (Wang et al., 2007; Tomlinson and Silva, 2015). The subcutaneous implantation of gelatin sponges soaked in human MSC conditioned medium in diabetic rats enhanced fracture healing, with increased bone formation and vessel ingrowth compared to control animals (Wang et al., 2012). Besides paracrine signaling, direct cell-cell interaction can also be involved in this cross talk. Functional gap junctions have been observed between osteoblasts and endothelial cells (Leszczynska et al., 2013). In this study, primary human osteoblasts were co-cultured with ECs, resulting in a four-fold increase in osteoblast numbers compared to osteoblast monocultures, suggesting a direct interaction that promotes osteogenic differentiation. A more recent study showed that human ECs in co-culture with human MSCs promoted their osteogenic differentiation (Genova et al., 2019). Conversely, osteo-differentiating MSCs enhanced angiogenesis and EC recruitment (Genova et al., 2019). Similar results were observed in a co-transplantation experiment of human osteogenic MSCs and endothelial progenitor cells in nude mice, which formed more dense bone with a higher vascular density compared to transplantation of osteogenic MSCs alone, suggesting that endothelial progenitor cells promote bone formation (Zhang et al., 2012). Moreover, there have been indications that human ECs transdifferentiate into osteoblasts in normal bone development, suggesting that ECs actively contribute to the process of bone development. However, the potential (significant) role of trans-differentiation of ECs in the context of fracture healing is yet to be discovered. All these findings combined strongly advocate for angiogenic-osteogenic coupling in the bone microenvironment, which is important to understand the process of uncompromised fracture healing.

## 3 Energy metabolism

Energy metabolism is an essential process in life to provide cells with energy and nutrients to meet their demands for homeostasis and growth. During fracture healing, cell proliferation, migration and differentiation within the fracture site are crucial for proper tissue regeneration. To generate cellular energy, adenosine 5′-triphosphate (ATP) is generated from carbohydrates, fatty acids and amino acids. Glucose is the main source of energy for most of the cell types, including stem cells. Glucose enters the cell via glucose transporters (GLUT), where it is first metabolized in the cytoplasm. Several successive enzymatic steps convert one glucose molecule into 2 pyruvate molecules, thereby producing 2 ATP moieties and 2 reduced molecules of nicotinamide adenine dinucleotide (NADH). In anaerobic conditions, pyruvate is converted into lactate in the cytoplasm, generating oxidator NAD+ to maintain the process of glycolysis during oxygen deprivation. In the presence of oxygen, pyruvate will be transferred into the mitochondria to be oxidized within the tricarboxylic (TCA) cycle. Electrons liberated from TCA cycle intermediates can generate ATP through oxidative phosphorylation (OxPhos). OxPhos generates a 15 times higher ATP yield compared to glycolysis. In addition to glucose, both amino acids and fatty acids can fuel the TCA cycle. One of the most abundant nonessential amino acids is glutamine, which can be converted to α-ketoglutarate to fuel the TCA cycle (Chandel, 2021). It is known that amino acids play a crucial role in successful fracture healing, as it was reported that amino acid concentrations in the callus of non-union patients are altered compared to patients with normal fracture healing (Wijnands et al., 2012). Moreover, it has been observed that physiological concentrations of short chain fatty acids, like (sodium) butyrate, have beneficial effects on osteogenic differentiation in fracture healing (Fan et al., 2018).

Fatty acids are broken down via the complex process of beta-oxidation (Kushwaha et al., 2018). During each cycle of beta-oxidation, two carbons are removed to form acetyl-CoA to fuel the TCA cycle. One cycle of beta oxidation has the potential to generate 17 ATP. As multiple rounds are conducted to break down a complete fatty acid molecule, it can produce significantly more ATP molecules compared to 36 ATP moieties resulting from complete dissimilation of one glucose molecule (Kushwaha et al., 2018).

Besides generating energy, metabolic intermediates are also used for anabolic processes, maintaining cellular homeostasis. Some cell types prefer glycolysis above OxPhos in the presence of oxygen, known as the ‘Warburg effect (Karner and Long, 2018). However, despite the inefficient way of producing ATP, the rate of ATP production from glucose is approximately 100 times faster compared to OxPhos (Shen et al., 2022). Hence, it is thought that aerobic glycolysis somehow facilitates rapid growth, as it is often seen in cancer cells. In this review, we describe the metabolic landscape of the main cells involved in fracture healing, and how energy metabolism potentially plays a role in the angiogenic-osteogenic coupling.

### 3.1 Osteoblast metabolism

The metabolic profile of human MSCs, osteoblasts and ECs has been previously studied. Stem cells in general rely primarily on glycolysis (Varum et al., 2011; Shum et al., 2016). Bone marrow-derived MSCs (BMSCs) generally reside in hypoxic areas, relying on glycolysis as well (Ning et al., 2022). As already mentioned, HIF1α is stabilized in hypoxic conditions. HIF1α stabilization in osteoblast precursor cells leads to the direct upregulation of glycolytic enzymes, driving proliferation and differentiation towards osteoblasts (Regan et al., 2014). This indicates that active glycolysis stimulates the development of osteoblasts. Moreover, Shum et al. investigated the bioenergetic profile of osteo-differentiating BMSCs. Upon in vitro differentiation, BMSCs activate OxPhos, while glycolysis is maintained at a similar rate as in their undifferentiated state (Shum et al., 2016). Interestingly, HIF1α was drastically downregulated in osteo-differentiating BMSCs compared to undifferentiated MSCs (Shum et al., 2016). This is in line with the fact that HIF1α downregulates OxPhos (Papandreou et al., 2006). Nonetheless, the downregulation of HIF1α does not affect the glycolytic rate in osteo-differentiating osteoblasts. It has been shown that fatty acid metabolism is essential for further osteoblast differentiation as the inhibition of CPT1A, a key enzyme in fatty acid oxidation, was shown to prevent differentiation into the osteoblast lineage in vivo (van Gastel et al., 2020). It is suggested that OxPhos activation during osteoblast differentiation is required to meet high ATP demands for processes such as extracellular matrix protein production. In line with this, Kim et al. found evidence that fatty acids, taken up by osteoblasts, are likely to be processed for ATP generation, as de novo fatty acid production in osteoblasts was low (Kim et al., 2017). Despite active use of OxPhos during differentiation, mature osteoblasts again mainly rely on glycolysis (Esen et al., 2013). Around 80 % of the glucose is metabolized into lactate, even in the presence of oxygen, known as the Warburg effect (Shen et al., 2022).

As most of the fractures heal via indirect healing, it is important to address the behavior of chondro-differentiating BMSCs. In general, chondroblasts and chondrocytes reside under hypoxia in vivo (Meleshina et al., 2017). It was observed that chondroblasts continue to rely on glycolysis during differentiation. Furthermore, low oxygen tension (5 %) significantly increased the rate of protein synthesis in chondrogenic conditions, which phenomenon is related to the Warburg effect (Wang et al., 2005). Moreover, the low extracellular lipid concentration in the early callus by the lack of vascular supply promotes differentiation towards chondrogenic differentiation by the activation of transcription factor SOX9 (van Gastel et al., 2020). Internal lipids are mobilized after vascular disruption, causing nuclear localization of FOXO that promotes expression of SOX9, inducing chondrogenic commitment (van Gastel et al., 2020). One explanation given for this adaptation to acute nutritional stress is the avoidance of cell death. Taken together, the process of endochondral ossification can continue independently from oxygen concentrations in the callus, whereas osteoblast differentiation relies more on oxygen and fatty acids. Furthermore, this data emphasizes that during the initial differentiation from BMSCs to osteoblasts, glycolysis is essential, whereafter OxPhos is activated to fulfill the energy demand during differentiation and ECM synthesis and to eventually switch back to glycolysis to maintain osteoblast functions.

### 3.2 Endothelial cell metabolism

Independent from oxygen levels, ECs prefer to generate ATP via glycolysis (Loeffler et al., 2018). In relation to this, ECs have a relatively small mitochondrial volume, which takes up approximately 2 to 6 % of the cell's volume (Oldendorf et al., 1977). Upon activation, the glycolytic rate is doubled compared to quiescent ECs (De Bock et al., 2013). Since ECs predominantly derive their ATP from glycolysis, this does not limit the ECs for sprouting in hypoxic areas, like the fracture callus. At the fracture site, ECs are activated to migrate and proliferate via VEGF signaling. VEGF signaling directly upregulates glycolytic enzymes to increase the glycolytic rate, including lactate dehydrogenase (LDH), GLUT1 and Phosphofructo-2-Kinase/Fructose-2,6-Biphosphatase 3 (PFKFB3) (De Bock et al., 2013). Despite the fact that ECs in general have a small mitochondrial volume, OxPhos is important for EC proliferation as well. ECs triple their oxygen consumption rate upon proliferation during angiogenesis (Eelen et al., 2018). Furthermore, ECs are able to adapt their metabolism to the microenvironment, as in conditions of glucose depletion ECs can switch to OxPhos using fatty acids and amino acids for ATP production (Loeffler et al., 2018). Limiting the fatty acid oxidation rate leads to decreased EC proliferation and sprouting defects. This is not likely to be caused by energy limitation, as approximately 5 % of the cellular ATP derives from OxPhos, but probably due to the lack of biosynthesized substrates generated by OxPhos in the nutrient-scarce environment and the consequential redox imbalance (Eelen et al., 2018). Thus, ECs primarily rely on glycolysis, but OxPhos activation is still essential during angiogenesis.

Altogether, despite the need for additional insights, the metabolic landscapes of cell types involved in fracture healing for different species are well described. As the (metabolic) microenvironment is such an important component for bone regeneration, it is suggested that metabolic coupling at the angiogenic-osteogenic interface supports successful fracture healing.

## 4 Metabolic coupling

**Fig. 1:**
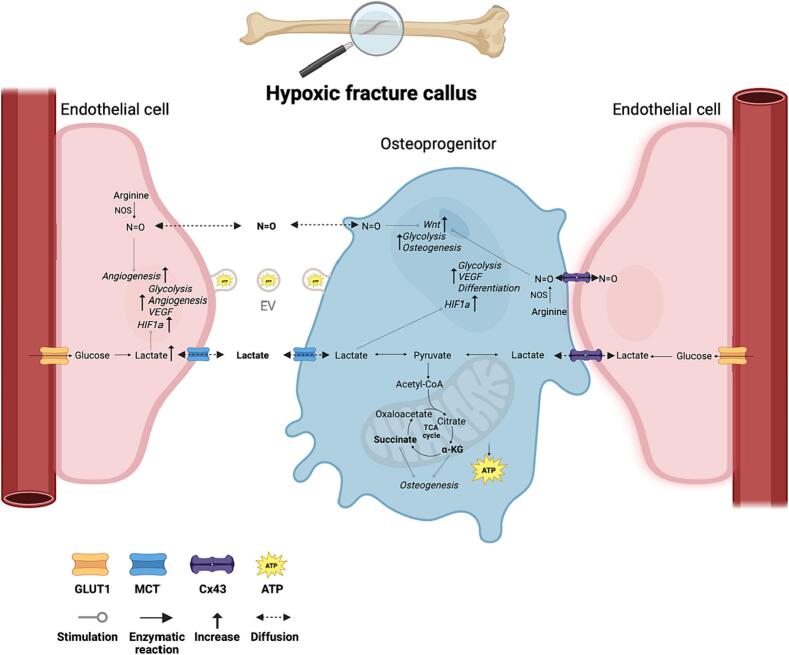
Schematic representation of the (metabolic) angiogenic-osteogenic interphase. ATP = adenosine 5′-triphosphate, Cx43 = Connexin 43, GLUT = glucose transporter, HIF1α = hypoxia inducible factor 1-alpha, α-KG = alpha-ketoglutarate, MCT = monocarboxylate transporter, N=O = nitric oxide, NOS = nitric oxide synthase, OxPhos = Oxidative Phosphorylation, VEGF = vascular endothelial growth factor.

Metabolic crosstalk is defined as the exchange of secreted factors that act through *endo*- and paracrine communication to alter metabolism and energy homeostasis. Amongst many other cell types, BMSCs and ECs in the fracture callus are exposed to secreted signaling molecules, including metabolites, thereby creating a complex and bidirectional metabolic coupling at the angiogenic-osteogenic interface (Fig. 1). Nevertheless, the mechanisms underlying this intricate metabolic crosstalk remain to be elucidated. Recently, researchers have focused on the metabolic profiles of human BMSCs and ECs in the process of osteogenic-angiogenic coupling. Petrillo et al. investigated the non-direct interaction of human dermal microvascular endothelial cells (HMECs) and human exfoliated deciduous teeth stem cells (SHEDs). SHEDs are used as model for BMSCs because of their similarities in mesenchymal origin, including their representative marker expression (Kashyap, 2015). HMEC condition medium (HMEC-CM) experiments revealed that anaerobic glycolysis is activated in proliferating SHEDs grown with HMEC-CM. Furthermore, HMEC-CM minimally influences the metabolic switch to OxPhos in osteo-differentiating SHEDs. These results show that endothelial cells promote glycolysis, giving stem cells the ability to proliferate in hypoxic areas, like the fracture callus, without compromising the ability of oxidative metabolism required for osteoblast differentiation (Petrillo et al., 2021). Conversely, Zhang et al. performed a transcriptomic profile analysis on human umbilical vein endothelial cells (HUVEC) cultured in a novel microfluidic chip with human BMSC-derived condition medium, reflecting the in vivo hypoxic and dynamic microenvironment in the process of angiogenesis. Genes involved in glycolysis, fatty acid metabolism and oxidative phosphorylation were all upregulated compared to the static culture and when using non-conditioned medium. This suggests that paracrine BMSC signaling enhances metabolism in ECs that exerts a positive effect on angiogenesis (Zhang et al., 2022). Although these results provide a good indication of bidirectional metabolic crosstalk, the process of fracture healing is very dynamic, which is also reflected by heterogeneous cell metabolism in the callus. Schilling et al. analyzed the metabolic state of cell types in vivo in murine cranial bone defect repair via two-photon fluorescence lifetime imaging microscopy (FLIM). This technique allows for the spatiotemporal analysis of the autofluorescence of free and bound NAD(*P*)H at single cell resolution, representing the metabolic state of the cell (Schilling et al., 2022a). The authors showed that ECs at the leading fracture site are in a glycolytic state, independent from the oxygen levels in the microenvironment, which is in line with previous studies (De Bock et al., 2013; Eelen et al., 2018). Furthermore, osteoblasts at the leading edge of repair were also in a more glycolytic state, which can be related to the low oxygen levels at the angiogenic-osteogenic interface. However, OxPhos is activated as well in the osteoblast in the fracture callus, suggesting that the limited oxygen is consumed by osteoblasts, and not by ECs. Hence, the expanding osteoblasts at the leading edge carry out aerobic glycolysis and OxPhos, which favors active proliferation and differentiation (Schilling et al., 2022b).

The illustration was made with BioRender.

### 4.1 Metabolic intermediates

As a result of active glycolysis in osteoblasts and ECs, a substantial amount of lactate is secreted in the early callus (Loeffler et al., 2018). Lactate has an important function to maintain redox homeostasis (Li et al., 2022). Moreover, lactate is not only an end product of glycolysis but can also be taken up by cells and be further metabolized within the TCA cycle (Hui et al., 2017). Therefore, taking the metabolic landscapes of BMSCs and ECs into account, it is feasible that lactate can contribute to fueling osteoblast differentiation at the leading fracture site. It is known that lactate can have several functions in the fracture callus. Lactate contributes to the angiogenic response by stabilizing HIF1α in ECs (Sonveaux et al., 2012). Moreover, lactate induces the migration of BMSCs (Rattigan et al., 2012). This study also emphasizes the metabolic cooperation between cell types, whereby one cell secretes lactate which is taken up via monocarboxylate transporter 1 (MCT1) in other cells to be used as a source of energy, which may also take place during fracture healing (Rattigan et al., 2012; Wu et al., 2017). Finally, there are indications that lactate can induce osteoblast differentiation by fueling OxPhos (Wu et al., 2017). A recent study has shown that lactate supplementation in mice trained with high-intensity interval training improves bone density and metabolism, which is hypothesized to be attributed to enhanced osteoblast differentiation (Zhu et al., 2023). These results support an important role of lactate in the fracture callus, and points towards a metabolic crosstalk of endothelial cells and osteoprogenitor cells through lactate.

Besides energy supply and source for anabolic processes, metabolic intermediates can also function as signaling molecules. Loeffler et al. have recently emphasized the role of succinate as metabolic communicator, steering successful bone regeneration. In addition to succinate, an upregulation of lactate secretion in the fracture callus was observed at day 7 of fracture healing compared to a condition with comprised healing (Loffler et al., 2023). Moreover, it has been suggested that α-ketoglutarate, another intermediate of the TCA cycle, can have pro-osteogenic signaling effects (Zurek et al., 2019). Altogether, there are several indications that metabolic intermediates play a crucial role at the cellular metabolic interphase, which is worth investigating further.

### 4.2 Nitric oxide

Another signaling molecule known to be involved in successful fracture repair is nitric oxide (NO) (Diwan et al., 2000). The gaseous and unstable molecule is very diffusible, as it can move easily inside cellular compartments and outside the cell. NO is produced from L-arginine by nitric oxide synthase (NOS), expressed in both ECs and osteoblasts (Diwan et al., 2000; Fox and Chow, 1998). Initially, NO released by ECs causes vasodilation in the inflammatory phase to increase the blood flow towards the fracture callus (Tomlinson et al., 2014). Hence, it has been shown that NOS and NO are essential in later stages of fracture healing, as suppression of NOS impairs fracture healing and NO supplementation can reverse this inhibitory effect (Diwan et al., 2000). As already mentioned, the amino acid concentrations in the callus of non-union patients are altered compared to patients with normal fracture healing, which can have an effect on the production of NO (Wijnands et al., 2012). Indeed, in non-union patients, the arginine concentration was decreased compared to patients with normal fracture healing. Moreover, the most abundant amino acid in the human body, glutamine, is known as an important nitrogen regulator inside the cell. It has been shown that glutamine supplementation has positive effects on fracture healing, mainly caused by an improved nitrogen balance (Polat et al., 2007). Recently, NO was identified as a promoter of osteogenesis via regulation of the Wnt pathway, which is vital for equine osteogenic differentiation (Bandara et al., 2016). The Wnt pathway is also involved in regulating energy metabolism by activating aerobic glycolysis in murine osteoblasts (Esen et al., 2013). Furthermore, Wnt signaling supports bone formation by enhancing glutamine catabolism though the TCA cycle in mouse osteoblasts (Karner et al., 2015). Previously, it was shown that NO enhances glycolysis in skeletal muscle cells (Stamler and Meissner, 2001). The role of NO on energy metabolism in osteoblasts has been investigated through conditional knockout mice for osteoblast-specific argininosuccinate lyase (Asl), which is the key enzyme to generate arginine intracellularly (Jin et al., 2021). Asl knockout mice show decreased NO production and impaired osteoblast differentiation, which was caused by the loss of NO-mediated glycolysis. Moreover, there are indications that NO may also promote mitochondrial oxygen consumption during osteoblast differentiation, as Asl deficiency leads to impaired oxygen consumption (Jin et al., 2021). Collectively, NO appears to play an important role in regulating energy metabolism during osteoblast differentiation. Therefore, it is conceivable that NO derived from endothelial cells enhances the osteogenic differentiation of BMSC at the angiogenic-osteogenic interface.

### 4.3 Extracellular vesicles

Another means of indirect cell-cell communication are extracellular vesicles (EVs) that can facilitate the exchange of proteins, lipids and (small) RNAs (Turchinovich et al., 2019). It has been shown that ATP-containing EVs are secreted from endothelial cells upon hypoxia (Lim To et al., 2015). Moreover, rat osteoblasts secrete ATP via EVs (also in hypoxic conditions), which influences the process of bone remodeling (Orriss et al., 2009). In addition to ATP, it has been shown that EVs derived from hMSCs contain proteins and metabolites, including lactic acid and glutamic acid (a derivative of glutamine) in hypoxic conditions (Vallabhaneni et al., 2015). These studies implicate a pivotal role of EVs and their cargo in the hypoxic environment during fracture healing.

### 4.4 Gap junctions

BMSCs, osteocytes and endothelial cells can directly interact by Connexin43 (Cx43) gap junctions (Villars et al., 2002; Lin et al., 2018). Gap junctions allow the passage of molecules smaller than 1 kD, including metabolites like lactate and glucose (Riquelme et al., 2020). Blocking these gap junctions delays the process of fracture healing in vivo, suggesting an important role in their communication during fracture healing (Villars et al., 2002). As osteoblast differentiation is decreased upon blocking gap junctions, this suggests that endothelial cells contribute to osteoblast differentiation and that gap junctions play an essential role in this communication. However, whether this is due to alteration of gene expression or lack of energetic fueling remains to be elucidated. Recently, it has been shown that MSCs and ECs can directly transfer metabolites as energy sources to enhance energy metabolism of other cells (Ogawa et al., 2022). Indeed, NAD+ can be released by MSCs via Cx43 gap junctions, causing positive autocrine effects on BMSC proliferation and migration (Fruscione et al., 2011). NAD+ is an important player in redox balance, so theoretically transferring NAD+ metabolites could influence metabolic fluxes. Although metabolite transfer through gap junctions between cells is likely to occur during fracture healing, its crucial role remains to be proven.

## 5 Concluding remarks

To conclude, energy metabolism plays a crucial role in successful fracture healing, contributing not only by providing intermediates for energy purposes but also by signaling towards differentiation. Glycolysis, amino acid and fatty acid metabolism all contribute to these processes. ECs likely carry out aerobic glycolysis while osteo-differentiating cells require OxPhos alongside glycolysis during the aftermath of a fracture. The lactate secreted at the angiogenic-osteogenic interface can possibly fuel osteoblast differentiation, optimally utilizing nutrients in the hypoxic and poor nutritional callus environment. Furthermore, lactate and NO can act as signaling molecule, promoting endothelial and osteoblast differentiation. Moreover, other metabolic intermediates, such as succinate and α-ketoglutarate, could possibly play a role regarding osteoblast differentiation. These molecules could be significant players in the metabolic crosstalk, although more research is mandatory to prove this. The communication of osteoblasts and ECs can be established through direct and indirect means, involving gap junctions and extracellular vesicles, respectively.

Besides the metabolic heterogeneity inside the fracture callus, the temporal healing pattern is essential to take into account. The initial crosstalk following a fracture could be significantly different from the crosstalk during the later stages of fracture healing. Hence, it is important to develop representative spatiotemporal in vitro bone fracture models. In vitro and ex vivo bone fracture models are becoming more advanced and optimized to better represent the in vivo fracture callus (Zhu et al., 2023; Loffler et al., 2023). The two-photon FLIM microscopy, by which real-time metabolic states of single cells in vivo can be measured, is a powerful tool for future scrutiny of spatiotemporal metabolic coupling during fracture healing.

Enabling effective treatment, for example by local supplementation of metabolites such as lactate in the fracture callus of patients with non-union fractures, will yield more insights into the angiogenic-osteogenic metabolic crosstalk and spatiotemporal processes that are essential during fracture healing, both in health and disease.

## Ethics approval and consent to participate

Not applicable.

## Consent for publication

Not applicable.

## Funding

Yudong Zhao is supported by the 10.13039/501100004543China Scholarship Council through a PhD Research Fellowship Grant (No. 202208510043).

## CRediT authorship contribution statement

**Fleur van Brakel:** Writing – review & editing, Writing – original draft, Visualization, Conceptualization. **Yudong Zhao:** Writing – review & editing, Writing – original draft. **Bram C.J. van der Eerden:** Writing – review & editing.

## Declaration of competing interest

The authors declare that they have no known competing financial interests or personal relationships that could have appeared to influence the work reported in this paper.

## Data Availability

No data was used for the research described in the article.
